# Automated Segmentation of the Systolic and Diastolic Phases in Wrist Pulse Signal Using Long Short-Term Memory Network

**DOI:** 10.1155/2022/2766321

**Published:** 2022-08-21

**Authors:** Lin Huang, Jianjun Yan, Shiyu Cai, Rui Guo, Haixia Yan, Yiqin Wang

**Affiliations:** ^1^Institute of Intelligent Perception and Diagnosis, School of Mechanical and Power Engineering, East China University of Science and Technology, Shanghai 200237, China; ^2^Shanghai Key Laboratory of Health Identification and Assessment, Comprehensive Laboratory of Traditional Chinese Medicine Four Diagnostic Information, Shanghai University of Traditional Chinese Medicine, Shanghai 201203, China

## Abstract

**Purpose:**

Single-period segmentation is one of the important steps in time-domain analysis of pulse signals, which is the basis of time-domain feature extraction. The existing single-period segmentation methods have the disadvantages of generalization, reliability, and robustness.

**Method:**

This paper proposed a period segmentation method of pulse signals based on long short-term memory (LSTM) network. The preprocessing was performed to remove noises and baseline drift of pulse signals. Thus, LabelMe was used to label each period of the pulse signals into two parts according to the location of the starting point of main wave and the dicrotic notch, and the dataset of the pulse signal period segmentation was established. Consequently, the labeled dataset was input into the LSTM for training and testing, and the results were compared with sum slope function method.

**Result:**

The remarkable result with the whole period segmentation accuracy of 92.8% was achieved for the segmentation of seven types of pulse signals. And the segmentation accuracies of the systolic phase, diastolic phase, and whole period using this method were higher than those of the sum slope function method.

**Conclusion:**

LSTM-based pulse signal segmentation method can achieve outstanding, robust, and reliable segmentation effects of the systolic phase, diastolic phase, and whole period of pulse signals. The research provides a new idea and method for the segmentation of pulse signals.

## 1. Introduction

Wearable pulse wave detection devices are more and more applied for health monitoring. The pulse signal contains very rich cardiovascular physiological and pathological information. According to the pulse diagnosis theory of traditional Chinese medicine (TCM), using the pulse signal can not only detect whether the subject is abnormal but also predict the pathological condition. Therefore, it is of great research significance to make an objective description of pulse signals based on the theory of TCM pulse diagnosis. In the study of the objectification of pulse diagnosis, pulse signal processing is a crucial step in obtaining the diagnosis result of Chinese medicine, including pulse signal segmentation and feature extraction and pulse signal pattern recognition and classification. Thus, accurate segmentation and feature extraction of pulse signals are very important for the objectification of pulse diagnosis. In practical applications, the pulse signal is extremely susceptible to external interference from the environment, individuals, acquisition equipment, etc., which will add different noises to the signal and make it difficult to segment.

There are many methods for pulse signal segmentation. Zong et al. [1] proposed an algorithm to effectively detect the starting point of the blood pressure wave, using windowed and weighted slope function to extract the characteristics of the blood pressure wave and adopting an adaptive threshold and search strategy to determine the starting point of the pulse wave. Xu et al. [2] proposed a neighbor pulse-based signal enhancement algorithm to detect pulse onset locations from noise-contaminated pulsatile signals, in which pulse onset was detected using the first principal component extracted from three adjacent pulses. It improved pulse onset detection according to all three different onset definitions. Yang et al. [3] presented an algorithm to identify the onset of intracranial pressure (ICP) pulses, which created a waveform descriptor to extract the feature of each local minimum of the waveform, and then identified the onset by comparing the feature with a customized template. Karlen et al. [4] developed a pulse segmentation algorithm using the derivative of the signal to find pulse slopes and an adaptive set of repeated Gaussian filters to select the correct slopes, and cross-correlation of consecutive pulse segments was used to estimate signal quality. Asgari et al. [5] utilized the adaptive thresholding method (ADT) for onset detection of intracerebral blood flow velocity (CBFV) pulse signals, resulting in improved performance levels of 93.1% and 93.3%, respectively. Ding et al. [6] applied a simple segmentation method in the derivative of pulse waves for segmentation, which regarded the first zero point of the derivative before each threshold point in all threshold points of the derivative was defined as the period segmentation point. Vadrevu and Manikandan [7] presented an automated robust multiscale sum and product (MSP) method for pulse peak and onset detection, which combined MSP, Shannon entropy envelope extractor, and Gaussian derivative-based peak finder by understanding the temporal-spectral characteristics of the PPG signals and the background noises and artifacts. Lee et al. [8] presented a pulse onset detection method based on the Monte Carlo approach. It applied the Monte Carlo simulation for calculating sampling frequency, and the position of a systolic peak which means a beat cycle could be accurately derived by a moving time window. Thus, the onset detection was robust from noise and artifacts of a signal. Oppenheim and Sittig [9] developed a dicrotic notch detection algorithm that combined the most appropriate method for accurate notch localization based on a series of extracted waveform features. Donelli et al. [10] evaluated an algorithm for real-time detection and prediction of the dicrotic notch from aortic pressure waves in arrhythmic aortic pressure signals. The dicrotic notch was detected at the first negative dip from the aortic flow calculated by a simplified model of the arterial tree, and prediction of the notch was performed using a percentage of the decreasing flow. Singh and Sunkaria [11] employed empirical wavelet transform for locating the systolic peak and onset of blood pressure pulse and utilized the first-order difference of blood pressure pulse for dicrotic notch detection. Balmer et al. [12] defined a new end systole detection algorithm for dicrotic notch-less arterial waveforms. The algorithm was adaptive based on previous heartbeat end systole locations, which employed beta distribution probability density function as a weighting function. However, the existing segmentation methods of pulse signals lack generalization, reliability, and robustness. Moreover, these methods require researchers to have professional knowledge in pulse wave signal processing.

As a new research direction in the field of machine learning, deep learning is becoming more and more widely used in medicine. Its representative network models include Convolutional Neural Network (CNN) and Recurrent Neural Network (RNN). LSTM improves the traditional RNN and has been successfully applied to ECG signal segmentation and has achieved remarkable results [13]. Therefore, this paper proposed a new segmentation method of the systolic and diastolic phases of pulse signals based on LSTM to improve the segmentation accuracy, reliability, and robustness.

## 2. Methods

### 2.1. Pulse Data Acquisition Equipment

**Figure 1:**
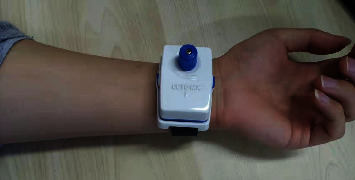
Pulse signal acquisition device.

In this paper, the pulse signal data for analysis is acquired using a portable pulse acquisition device (product by Shanghai Asia & Pacific Computer Information System Co., Ltd., Shanghai, China) shown in Figure 1. The device is composed of a pulse sensor, a hardware circuit board, and a software analysis system. The pulse signal obtained from the pulse sensor by pressing the radial artery site is conditioned and amplified by the hardware circuit board and then processed in the computer software analysis system through the USB interface.

### 2.2. Time-Domain Characteristics of the Pulse Signal and Segmentation Task

The formation of the pulse signal is due to the regular systole and diastole of the heart. The initial pulse wave is formed on the side of the aorta close to the heart, and it also starts from the aortic root, propagating the periodic signal changes caused by the heartbeat along the artery, so that it can influence the entire arterial system [14]. In this process, the pulse wave will be periodically affected by the cyclical systole and diastole of the heart. It is also fed back on the physiological information of the arteries at all levels. It can be used to measure information including blood vessel information, blood flow information, cardiovascular disease information, and even physiological information. Therefore, it can be said that the pulse signal is a combination of the initial pulse wave from the source to the downstream and the reflected wave from the downstream to the source. If the pulse signals containing a large amount of human physiological and pathological information can be properly analyzed, it will assist the doctor to diagnose and even to predict the disease of the patient.

**Figure 2:**
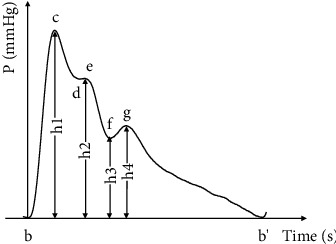
Schematic diagram of a typical pulse signal.

In order to analyze the pulse signal and to mine the pathological information it contains, it is also necessary to have a certain understanding of the characteristics of the typical pulse signal. The following will mainly introduce the characteristics of the typical pulse signal shown in Figure 2.

A relatively complete pulse wave period consists of an ascending branch and a descending branch, including four characteristic points. At the peak of the ascending branch, there is the main wave crest (point *c*), and then, there are three characteristic points in the descending branch, corresponding to the peak of the dicrotic wave (point *e*), the trough of the descending isthmus (point *f*), and the dicrotic wave peak (point *g*). For most people, points *c* and *g* are more obvious and can be easily identified.

- Ascending branch: it is starting from point *b* to point *c* of the main wave peak, reflecting that the pressure on the side of the aorta close to the heart has risen sharply
- Main wave: point *c* is the peak of the main wave, which means the maximum value of aortic pressure and blood volume, and represents the balance of the ventricular ejection and outflow at this time
- Descending branch: the part from point *c* to point *d* reflects that the ventricular ejection velocity begins to decrease, and the ventricle enters a slow ejection period
- Dicrotic prewave: it is formed by a reflected wave propagating towards the heart superimposed on the descending branch of the pulse wave, and point *e* is the peak of the dicrotic prewave
- Dicrotic notch: it is the point *f* of the trough representing the critical state of systole and diastole of the heart at this time
- Dicrotic wave: point *g* is at the peak of the dicrotic wave; blood pressure has slightly increased due to the closure of the aortic valve and aortic elastic retraction. Because it is clear enough, the presence of a dicrotic wave can be regarded as the important information for clinical diagnosis

The main task of pulse signal segmentation is single-period extraction of pulse signals. Point *f* where the dicrotic notch is located is taken as the demarcating point, and then, the single-period pulse signal is divided into systolic phase and diastolic phase, so as to facilitate the accurate extraction of the time-domain characteristics of the subsequent pulse signal.

### 2.3. LSTM-Based Pulse Signal Segmentation

**Figure 3:**
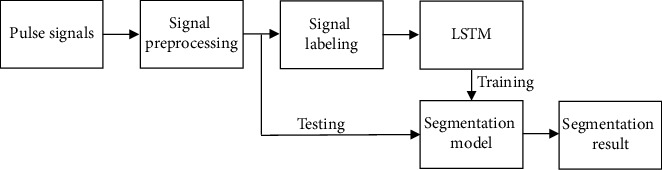
Pulse signal segmentation process.

LSTM was used to segment ECG signals in different stages, and remarkable results have been achieved. Both the pulse signal and the ECG signal belong to the human body's physiological signal and have similarities. Therefore, this article applied LSTM to segment pulse signals. The process of pulse signal segmentation is shown in Figure 3. Firstly, pulse signals were performed preprocessing to remove noises and baseline drift; secondly, pulse signals were manually labeled; thirdly, the LSTM model was built for training and testing.

### 2.4. Preprocessing of the Pulse Signal

**Figure 4:**
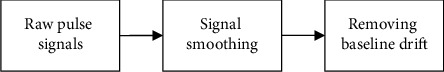
Preprocessing process.

The human pulse signal is a kind of nonstationary random signal with low signal-to-noise ratio. The frequency distribution of the signal is in the range of 0 to 40 Hz and is mainly concentrated below 20 Hz. In the process of acquisition, some useless signals and noises will be generated due to the interference of human respiration, acquisition equipment, working current, and other factors, so that the direct analysis of the original signal may not achieve satisfactory results. Therefore, pulse signals should be preprocessed before signal analysis and feature extraction according to the procedure shown in Figure 4. Pulse signal preprocessing includes signal smoothing and removing baseline drift.

**Figure 5:**
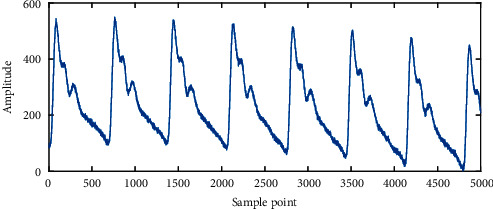
Pulse wave with noise.

Due to the influence of 50 Hz AC current and unstable acquisition work, the original pulse signal contains a lot of high-frequency noise. As shown in Figure 5, the pulse waveform with noises is not smooth and has a large number of burrs. Smooth processing using zero phase low-pass filter is employed to remove the frequency components above 20 Hz in the signal to reduce the noise.

**Figure 6:**
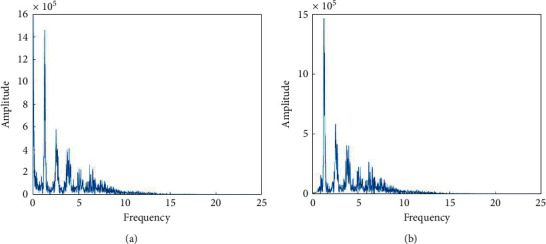
Spectrum comparison before (a) and after (b) removing baseline drift.

Due to the interference of human respiration, the original pulse signal produces baseline drift. The respiratory rate of the average person is not less than 12 times and not more than 24 times per minute; that is, the respiratory rate is less than 0.4 Hz. Signals in this frequency range can be removed by wavelet decomposition. After decomposing the pulse signal in 10 layers using sym8 wavelets, the wavelet coefficients from 0 to 0.35 Hz in the frequency band are cleared to zero, and then, the signal is reconstructed; thus, the frequency components below 0.35 Hz are removed. The frequency spectrum of a pulse signal before and after the processing is shown in Figure 6.

### 2.5. Pulse Signal Labeling

**Figure 7:**
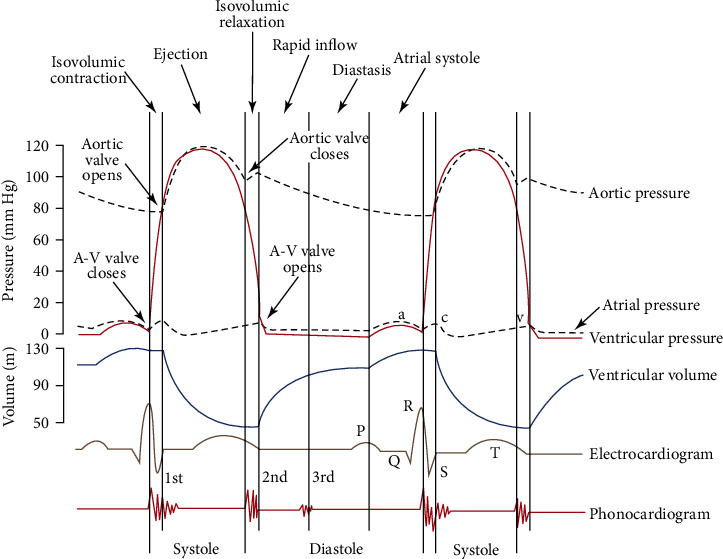
Blood pressure changes during the cardiac cycle [15].

The period segmentation of pulse signal is the basis of pulse signal analysis. Pulse waves are formed by the periodic beats of the heart. As shown in Figure 7, when the heart begins to contract, the aortic valve opens, and the increase in atrial pressure injects the remaining blood into the ventricle, which further increases the ventricular filling, and there is a small increase in ventricular pressure. At this time, the pulse wave is in the ascending stage, until the peak value of the main pulse wave is reached in the rapid ejection stage. Before the slow ejection and the heart begins to diastole, the pulse wave is generally in the downward branch. However, the characteristics of the disturbance by different factors are not obvious in different people, some have a direct decline, and others have a short rising phase. Until the aorta closes, heralding the beginning of the diastolic phase, this turning point corresponds to the descent of pulse waves into the middle isthmus. Thereafter, the heart diastolic causes the intra-atrial pressure to decrease and the blood to flow back until the start of the next isovolumic contraction, which completes a cardiac period. When the heart is in cyclic contraction and relaxation, the blood vessels can be palpated at the site of superficial vessels such as the radial artery that are constantly pulsating in sync with the heartbeat. A pulse wave is produced with one systolic and diastolic act of the heart, and the average systolic and diastolic duration of the ventricles is 0.27 and 0.53 seconds, respectively.

The determination of the systolic and diastolic phases is important for the time-domain analysis of the pulse signals. For normal and slippery pulses, the systolic and diastolic phases are easily determined by the dicrotic notch; for string-like pulses and other pulses, the dicrotic wave is not obvious or even disappears, so the position of the dicrotic notch is inconvenient to determine. Thus, this makes it difficult to determine the systolic and diastolic phases, which brings inconvenience to the time-domain feature extraction of the pulse signal. Therefore, the systolic and diastolic phases are manually labeled separately, using the dicrotic notch of the pulse wave as the boundary of the two phases.

There are less common labeled datasets of the pulse wave signal, and the labeling of the one-dimensional signal is difficult. In order to facilitate the labeling of pulse signals, pulse signals are converted into images and LabelMe is employed for labeling pulse signals in this paper. Considering a sufficient number of periods for segmentation in the pulse signal and the computational efficiency of the segmentation model, the length of the original signal is set as 2160 based on the sampling frequency of 720 Hz. And Matlab is used to convert the pulse signal into a waveform image with a resolution of 2264 × 1296. Therefore, the human-annotated positions in the signal are calculated based on the relationship between the width of the image and the actual signal length, as in the following formula:

(1) $$\begin{array}{c} S_{i}=\frac{P_{i}}{W}\times L\;\left(i=1,2,\cdots ,n\right), \end{array}$$

where *S*_*i*_ is the position of the labeled signal in the actual; *P*_*i*_ is the horizontal position in the waveform image; *W* is the image width; and *L* is the length of the original signal.

**Figure 8:**
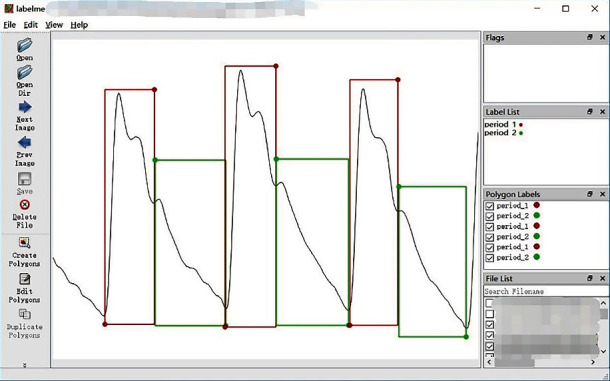
Labeling process.

LabelMe is used to label each period of the signal into two parts according to the location of the starting point of the main wave and the dicrotic notch. After the labeling, the results are saved into the JSON format file to establish the dataset of pulse signal period segmentation. The labeling process using LabelMe is shown in Figure 8.

### 2.6. Bidirectional LSTM

Recurrent Neural Network (RNN) is a common model for deep learning in video processing, text generation, language modeling, image processing, etc. It is the more suitable network structure for processing sequence-type data [16]. In the case of short sequences, RNNs are perfectly capable of predicting sequences, but in more complex scenarios, gradient vanishing or exploding is naturally inevitable. Vanishing gradients make it difficult to learn and tune the parameters of the earlier layers in the network, while exploding gradients make the learning unstable.

**Figure 9:**
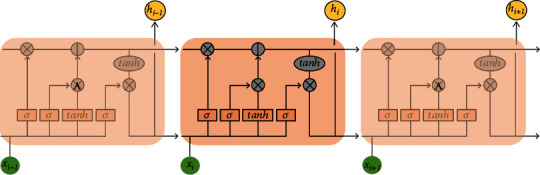
LSTM internal architecture.

Therefore, Hochreiter and Schmidhuber developed the LSTM model to solve this problem [17]. Its neural unit structure is shown in Figure 9. This model introduces the concept of gates, and gate structures such as “forget gates, update gates” can be used to decide whether information can pass through the gate to the next module or whether it should be retained or discarded directly, in order to ensure the forward transmission of valid data.

Bidirectional LSTM [18] is an upgrade of the one-way structure, which consists of a forward LSTM and a reverse LSTM. Compared with one-way LSTM, the sequence processing is more targeted and better in specific situations. Therefore, this paper employed bidirectional LSTM to build the period segmentation model of pulse signals.

**Figure 10:**
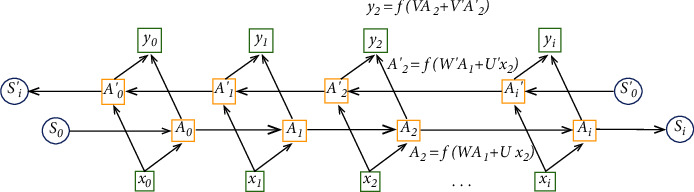
Bidirectional LSTM schematic.

The specific structure is shown in Figure 10, where a module is involved in the computation of the forward LSTM, while *A*′ is involved in the computation of the inverse LSTM.

LSTM is designed for time series modeling and overcomes the “gradient disappearance or explosion” problem in backpropagation when the dependencies are too long. In this paper, two bidirectional LSTM layers are constructed to constitute the feature extraction layer. One LSTM layer works as an encoder, and the other LSTM layer works as a decoder. For the LSTM unit as an encoder, the output number is the same as the function number. For the LSTM unit as a decoder, the output number is the same as the pulse signal length in this article. Specifically, the pretreated pulse signal *x*_*i*_ is used as the input of the LSTM model, and the output signal *y*_*i*_ is reconstructed from the input *x*_*i*_ through the LSTM model.

### 2.7. Experimental Setup

The dataset required for this paper is from the Comprehensive Laboratory of Traditional Chinese Medicine Four Diagnostic Information of Shanghai University of Traditional Chinese Medicine. There are 1400 cases of sample data, including 120 normal pulses, 100 slippery pulses, 360 fine and slippery pulses, 100 string-like and slippery pulses, 100 string-like pulses, 100 fine pulses, and 520 fine and string-like pulses. To facilitate the training and testing of the LSTM, the training set, validation set, and test set are divided according to the ratio of 8 : 1 : 1.

Python 3.8 is used as the development platform and Torch 1.10 is employed as the deep learning framework. The segmentation model is trained on a workstation having Intel Xeon Gold CPU with 128 GB RAM and Nvidia RTX A6000 with 48 GB memory. An Adam optimizer is used with an initial learning rate of 0.001.

## 3. Results

**Figure 11:**
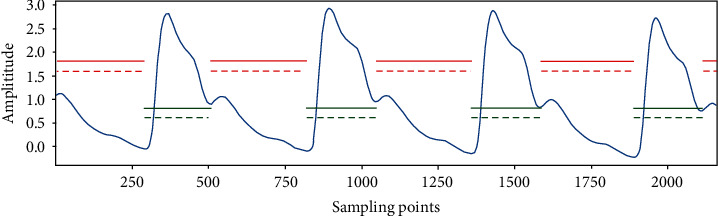
LSTM test results.

The experiment was performed on the test set using the LSTM segmentation model of the pulse signal, and the segmentation results are shown in Figure 11 and Table 1. The labeling and segmentation results of the systolic and diastolic phases are indicated by two line segments in Figure 11. The line segment on the upper side indicates the original manually labeled labels, and the dashed line segment on the lower side indicates the test results based on the LSTM model. The red dashed line represents the prediction of the systolic phase, and the green dashed line represents the prediction of the diastolic phase. The experimental results are in agreement with the original manually labeled results of the systolic phase and diastolic phase.

In order to further verify the effectiveness of the method proposed in this paper, the seven types of pulse signals in the test set are segmented separately and compared with the sum slope function- (SSF-) based signal segmentation method to calculate the segmentation accuracies of systole, diastole, and whole period of the pulse signals.

As can be seen from Table 1, the average accuracy of LSTM-based pulse signal segmentation was 94.6% in systole, 95.7% in diastole, and 92.8% in whole period, which were 7.2%, 7.1%, and 3.8% higher than that of the SSF, respectively.

## 4. Discussion

As can be seen from the introduction of the pulse waveforms mentioned above, the systole waveform generally consists of a main wave and a dicrotic prewave, and the diastole waveform contains a dicrotic wave. The systole waveform is more complex than the diastolic waveform, so the segmentation results of the pulse signal show a higher accuracy of the diastolic segmentation than those of the systolic segmentation. In order to investigate more closely the effectiveness of different methods for the segmentation of different kinds of pulse, the segmentation accuracies of seven types of pulse signals are compared and analyzed, and the results are shown in Table 2.

As can be seen from Table 2, the whole period segmentation accuracies of seven types of pulse signals are 94.7%, 94.9%, 94.8%, 93.2%, 89.5%, 91.4%, and 91.3%, which are 4.4%, 5.0%, 4.8%, 3.6%, 0.6%, 4.5%, and 4.3% higher than those of the SSF, respectively. The segmentation accuracies of diastolic and systolic phases of all seven types of pulse signals based on LSTM are also higher than those of SSF, which verifies the effectiveness of pulse segmentation based on the LSTM model in this paper.

**Figure 12:**
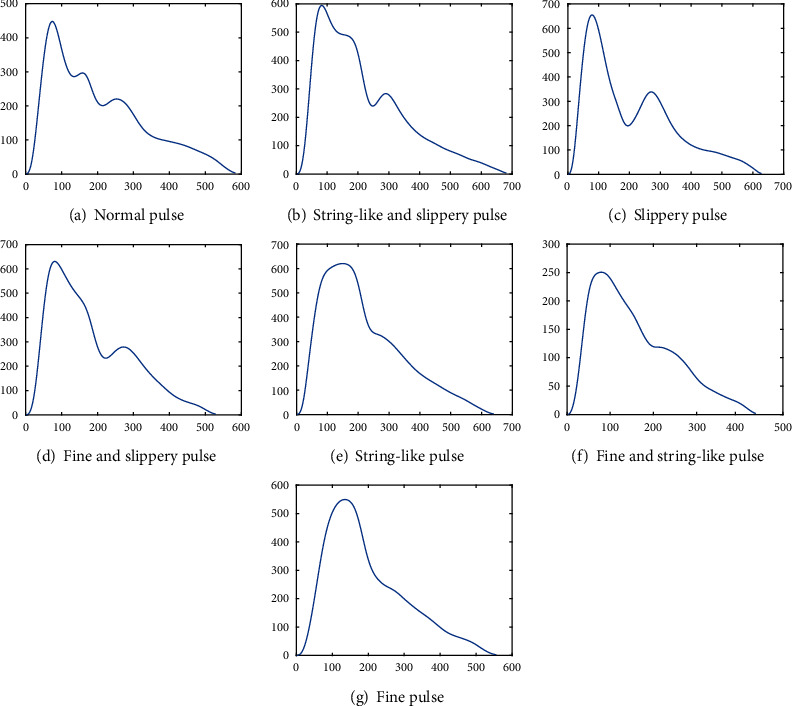
Seven types of pulse signals.

In order to further analyze the factors affecting the accuracy of pulse signal segmentation, the waveform diagrams of the seven types of pulse signals were analyzed. As shown in Figure 12, it can be found that the normal pulse is a three-peak pulse signal with a steep ascending branch and sharp and towering peaks of the main wave, and the clear dicrotic prewave and dicrotic wave are arranged in succession with the main wave, sloping down section by section. Therefore, the normal pulse is easier to segment through the dicrotic notch, so the systolic segmentation accuracy of the normal pulse is 96.1%, which is 7.2% higher than the SSF, while the diastolic segmentation accuracy is 96.2%, which is 6.2% higher than the SSF.

The string-like and slippery pulse, slippery pulse, and fine and slippery pulse are mostly bimodal pulse signals, similarly in morphological characteristics. The three types of pulse have a more obvious dicrotic wave, in which descending middle isthmuses are clearer, so that the locations of the dicrotic notch are also easy to determine. The systolic segmentation accuracy of the three types of pulses was 96.1%, 95.5%, and 95.4%, respectively; the diastolic segmentation accuracy was 96.7%, 96.7%, and 95.8%, respectively. For the three pulse signals, LSTM was 7.6%, 6.6%, and 7.4% more accurate than SSF in systolic segmentation, respectively, and 7.4%, 7.0%, and 6.8% more accurate in diastolic segmentation, respectively. Therefore, the pulse signal segmentation method proposed in this paper achieved better results than the SSF in the systolic and diastolic segmentation accuracies of string-like and slippery pulse, slippery pulse, and fine and slippery pulse.

The ascending and descending branches of string-like pulse, fine and string-like pulse, and fine pulse show sharp inclinations, and the dicrotic prewave appears in advance and coincides with the main wave, which is not obvious or even disappeared, resulting in the formation of a “flat top” type waveform. Moreover, the position of dicrotic notch is not obvious, and the systolic and diastolic phases of pulse could not be clearly distinguished. For these three types of pulse signals, the systolic segmentation accuracy of the proposed method is 93.4%, 92.9%, and 92.4%, and the diastolic segmentation accuracy is 93.6%, 94.9%, and 95.8%, respectively. For these three types of pulse signal waveforms with inconspicuous dicrotic notch locations, the LSTM segmentation accuracies are 5.5%, 7.9%, and 7.8% higher than SSF for systolic segmentation, respectively, and 5.2%, 8.2%, and 9.1% higher for diastolic segmentation, respectively. The segmentation method of pulse signals proposed in this paper is higher than SSF in the systole, diastole, and period segmentation accuracies of string-like pulse, fine and string-like pulse, and fine pulse and achieved more satisfactory results.

In conclusion, the pulse signal segmentation method proposed in this paper achieved good segmentation effects of the systolic phase, diastolic phase, and whole period. LSTM, as a special RNN, can solve the problem of gradient disappearance and gradient explosion in the training process of long sequences. Moreover, the feature engineering of the LSTM algorithm can be employed to effectively self-learn the pulse signals, and the features of pulse signals do not need to be manually extracted. Consequently, it may overcome the disadvantage of pulse signal segmentation by using only signal processing method to a certain extent.

## 5. Conclusion

Period segmentation is the basis of pulse signal analysis and recognition, so this paper proposed a period segmentation method of pulse signal based on LSTM. Firstly, pulse signals were performed preprocessing to remove noises and baseline drift; secondly, LabelMe was used to label 1400 cases of seven types of pulse signals manually; thirdly, LSTM was applied to establish the segmentation model of pulse signals, and training and testing were carried out; finally, the segmentation results of seven pulse signals were evaluated and analyzed. The results showed that this method can improve the accuracy of pulse signal segmentation to a certain extent. Therefore, the pulse signal segmentation method based on LSTM proposed in this paper provides a new idea and method for the period segmentation of pulse signals and has a certain practical application value for the analysis of pulse signals.

## Figures and Tables

**Table 1:** Comparison results of pulse signal segmentation (%).

Segmentation accuracy	LSTM	SSF
Systolic phase	94.6	87.4
Diastolic phase	95.7	88.6
Whole period	92.8	89.0

**Table 2:** Segmentation accuracy of seven types of pulse (%).

Method	Seven types of pulse	Segmentation accuracy		
		Systolic phase	Diastolic phase	Whole period
LSTM	Normal pulse	96.1	96.2	94.7
	String-like and slippery pulse	96.1	96.7	94.9
	Slippery pulse	95.5	96.7	94.8
	Fine and slippery pulse	95.4	95.8	93.2
	String-like pulse	93.4	93.6	89.5
	Fine and string-like pulse	92.9	94.9	91.4
	Fine pulse	92.4	95.8	91.3

SSF	Normal pulse	88.9	90.0	90.3
	String-like and slippery pulse	88.5	89.3	89.9
	Slippery pulse	88.9	89.7	90.0
	Fine and slippery pulse	88.0	89.0	89.6
	String-like pulse	87.9	88.4	88.9
	Fine and string-like pulse	85.0	86.7	86.9
	Fine pulse	84.6	86.7	87.0

## Data Availability

Due to the nature of this research, participants of this study did not agree for their data to be shared publicly, so supporting data is not available.
